# Paeonol-Loaded PLGA Nanoparticles Attenuate DMH-Induced Colorectal Carcinogenesis-Associated Oxidative Stress, Inflammation, and Cellular Dysregulation via Modulation of NRF2/HO-1 Signaling in Rats

**DOI:** 10.3390/ijms27135673

**Published:** 2026-06-23

**Authors:** M. Alfawaz, Ekramy M. Elmorsy, Ahmad Najem Alshammari, Eida M. Alshammari, Mai A. Salem, Gehad E. Elshopakey, Manal S. Fawzy, Nagwa M. Aly

**Affiliations:** 1 Department of Medical Laboratory Technology, College of Applied Medical Sciences, Northern Border University, Arar 91431, Saudi Arabia; mohammed.alfawaz@nbu.edu.sa (M.A.); ahmad.alshammari2@nbu.edu.sa (A.N.A.); 2 Center for Health Research, Northern Border University, Arar 73213, Saudi Arabia; ekramy.elmorsy@nbu.edu.sa; 3 Department of Chemistry, College of Sciences, University of Ha’il, Ha’il 2440, Saudi Arabia; eida.alshammari@uoh.edu.sa; 4 Department of Medical Biochemistry and Molecular Biology, Faculty of Medicine, Mansoura University, Mansoura 35516, Egypt; maisalem@mans.edu.eg; 5 Department of Clinical Pathology, Faculty of Veterinary Medicine, Mansoura University, Mansoura 35516, Egypt; gehadelshopakey@mans.edu.eg; 6 Department of Medical Biochemistry and Molecular Biology, Faculty of Medicine, Suez Canal University, Ismailia 41522, Egypt; nagwaaly@med.suez.edu.eg

**Keywords:** paeonol, PLGA nanoparticles, colorectal cancer, DMH model, NRF2/HO-1 signaling, oxidative stress, inflammation, apoptosis, autophagy, chemoprevention

## Abstract

Colorectal cancer (CRC) is driven by oxidative stress, chronic inflammation, and disruption of cytoprotective signaling pathways. This study aimed to evaluate whether poly(lactic-co-glycolic acid) (PLGA)-based nanoparticle delivery enhances the chemoprotective efficacy of paeonol against 1,2-dimethylhydrazine (DMH)-induced colorectal carcinogenesis, with a focus on modulation of the NRF2/HO-1 pathway. Sixty male Wistar rats were randomly assigned to six groups: control, paeonol (PNL), PNL-PLGA, DMH, DMH + PNL, and DMH + PNL-PLGA. CRC was induced using DMH over 10 weeks. Serum tumor biomarkers (AFP, CEA, CA19-9, CA125, CA15-3), oxidative stress markers (ROS, MDA, antioxidant enzymes), inflammatory cytokines, DNA damage, apoptosis- and autophagy-related gene expression, and hepatic and renal function were assessed. Histopathological and ultrastructural analyses of colonic tissues were performed. DMH exposure was markedly associated with increased tumor biomarkers, oxidative stress, and inflammatory mediators, DNA damage, and impaired liver and kidney function. It was also associated with the restoration of NRF2/HO-1 signaling, improved redox balance, suppression of inflammation, reduction in DNA damage, and preservation of regulated NRF2/HO-1 signaling, antioxidant defenses, autophagy markers, and apoptotic proteins, as well as severe histological and ultrastructural alterations. Free paeonol partially attenuated these changes. In contrast, PNL-PLGA was significantly associated with restoring NRF2/HO-1 signaling, improving redox balance, suppressing inflammation, reducing DNA damage, and preserving colonic architecture and ultrastructure. These findings demonstrate that a PLGA-based nanoformulation of paeonol markedly improves its chemopreventive efficacy against DMH-induced CRC, primarily by activating NRF2/HO-1 signaling and modulating oxidative stress, inflammation, apoptosis, and autophagy, highlighting its potential as a promising nanotherapeutic strategy for colorectal cancer.

## 1. Introduction

Colon cancer is one of the most frequent malignancies worldwide and remains a leading cause of cancer-related morbidity and mortality, particularly in industrialized countries [1]. Its development is multifactorial and reflects a complex interplay between environmental exposures, dietary habits, genetic predisposition, and lifestyle-related risk factors [2,3]. Established risk factors include high consumption of red and processed meats, low dietary fiber intake, obesity, physical inactivity, smoking, alcohol consumption, and chronic inflammatory bowel diseases [4]. Aging and inherited genetic mutations further contribute to disease susceptibility [5]. Among the molecular routes to colorectal tumorigenesis, the chromosomal instability pathway is considered the predominant genetic mechanism, characterized by large-scale chromosomal alterations, aneuploidy, and loss of heterozygosity [6]. Additional mechanisms further drive colorectal carcinogenesis, including chronic inflammation, microsatellite instability, aberrant DNA methylation, and dysregulated microRNA expression [7,8]. Together, these changes culminate in profound disruption of signaling networks governing cell proliferation, differentiation, apoptosis, and tissue homeostasis.

A key pathway involved in cellular defense against oxidative and electrophilic stress is the nuclear factor erythroid 2-related factor 2/heme oxygenase-1 (NRF2/HO-1) signaling axis [9]. NRF2 is a redox-sensitive transcription factor that regulates the expression of a broad array of antioxidant and cytoprotective genes, including HO-1, which plays a central role in maintaining cellular homeostasis and limiting inflammation and oxidative injury [10]. Under pathological conditions such as colorectal carcinogenesis, dysregulation of NRF2/HO-1 signaling contributes to excessive oxidative stress, impaired detoxification capacity, and enhanced tumor progression [11]. Modulation of this pathway has therefore emerged as a promising strategy for cancer prevention and therapy [12,13].

Experimental models are indispensable for dissecting the pathogenesis of colon cancer and for evaluating novel chemopreventive or therapeutic strategies. In this context, 1,2-dimethylhydrazine (DMH) is widely used as a potent chemical inducer of colon carcinogenesis in rodents [14]. Following administration, DMH undergoes hepatic biotransformation to reactive intermediates such as methylazoxymethanol and azoxymethane [15]. These metabolites ultimately generate the highly reactive methyl-diazonium ion, which alkylates DNA bases and initiates mutagenic events [16]. After metabolic activation in the liver, DMH-derived metabolites enter the systemic circulation, can be further processed in extrahepatic tissues, and are cleared via the kidneys, resulting in widespread tissue exposure and potential renal involvement in carcinogenesis [17]. In the colon, these metabolites induce DNA alkylation, trigger oxidative stress, and promote a sustained inflammatory response in epithelial cells [18]. Prolonged DMH administration leads to the formation of aberrant crypt foci, adenomas, and ultimately adenocarcinomas that closely resemble human colorectal cancer at histological and molecular levels [19].

Despite advances in conventional treatment modalities, including surgery, chemotherapy, radiotherapy, immunotherapy, and dietary interventions, the clinical management of colon cancer remains challenging. Treatment failure is often related to substantial adverse effects, incomplete tumor control, and the rapid emergence of drug resistance driven by underlying genetic and epigenetic instability [20]. Consequently, there is a continued need to develop alternative approaches that offer improved efficacy with reduced toxicity. In recent years, natural bioactive compounds have attracted considerable interest as potential anticancer agents because they typically act on multiple molecular targets, display favorable safety profiles, and can modulate key signaling pathways involved in tumor initiation and progression, including redox-sensitive pathways such as NRF2/HO-1 [21].

Paeonol (PNL; 2′-hydroxy-4′-methoxyacetophenone) is the main bioactive constituent of Moutan Cortex [22]. Accumulating evidence shows that paeonol exerts a broad spectrum of pharmacological effects, including antioxidant, anti-inflammatory, anti-proliferative, and anticancer activities [11,23]. Paeonol has demonstrated marked antitumor potential in a variety of cancer models in vitro and in vivo, where it can induce apoptosis, promote cell cycle arrest, and inhibit angiogenesis [23,24]. Its relatively low toxicity and favorable chemical structure make it an attractive candidate for further development as a targeted anticancer agent [25]. However, the clinical translation of paeonol is hampered by its poor aqueous solubility, limited oral bioavailability, and rapid metabolic degradation, all of which markedly constrain its therapeutic utility [26].

Nanotechnology-based drug delivery systems have been explored to overcome such limitations by enhancing drug stability, solubility, and site-specific delivery. Among available nanocarriers, poly(lactic-co-glycolic acid) (PLGA) nanoparticles have gained particular attention due to their biodegradability, biocompatibility, and regulatory approval for clinical use [27]. PLGA nanoformulations can improve drug bioavailability, prolong systemic circulation, and increase accumulation of the therapeutic payload at target tissues [27,28]. These advantages are often accompanied by reduced off-target toxicity and an improved therapeutic index [28]. In colorectal cancer, PLGA-based nanocarriers have been proposed as versatile platforms to address both bioavailability and resistance issues by enabling controlled release and more precise modulation of tumor-associated signaling pathways, including oxidative stress-related pathways such as NRF2/HO-1 [27,28].

In light of these considerations, the present study investigates paeonol-loaded PLGA nanoparticles (PNL-PLGA) as a potential nanotherapeutic strategy in a DMH-induced colon carcinogenesis model in rats. To the best of our knowledge, this is the first work to evaluate paeonol incorporated into a PLGA-based nanosystem in this experimental setting and to directly compare its efficacy with that of the free (non-nano) form. We assessed their ameliorative potential by focusing on major pathological processes that contribute to colorectal cancer progression, namely oxidative stress, inflammation, apoptosis, and autophagy, together with associated signaling alterations, particularly NRF2/HO-1 pathway modulation, to provide mechanistic insight into its enhanced chemopreventive efficacy.

## 2. Results

### 2.1. Physicochemical Characterization of PNL-PLGA Nanoparticles

**Figure 1:**
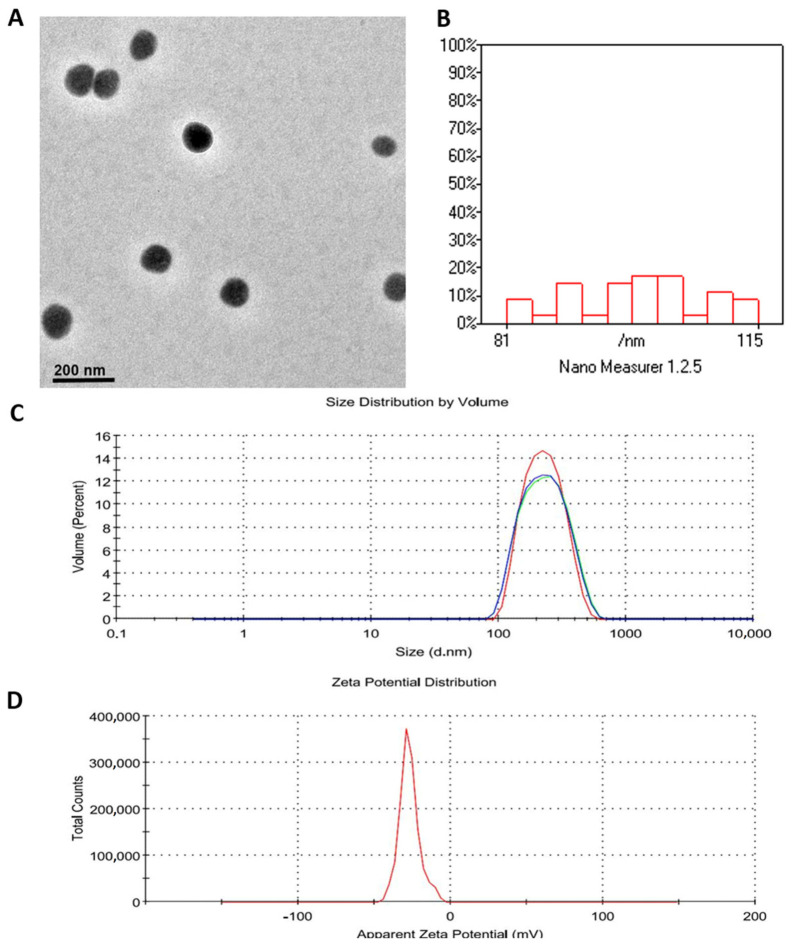
(**A**) Transmission electron microscopy image of paeonol-loaded PLGA (PNL-PLGA) nanoparticles. (**B**) Particle size distribution (number-based) showing the majority of particles between 81 and 115 nm. (**C**) Dynamic light scattering size distribution by volume for paeonol-loaded PLGA nanoparticles, showing overlaid size distribution curves from repeated measurements (red, green, and blue traces) with a narrow peak centered around ~100 nm and low polydispersity. (**D**) Zeta potential profile of PNL-PLGA nanoparticles.

Transmission electron microscopy (TEM) showed that paeonol-loaded poly(lactic-co-glycolic acid) nanoparticles (PNL-PLGA) were approximately spherical, well separated, and free of visible aggregation (Figure 1A). The particle size distribution histogram showed that most particles ranged from 81 to 115 nm, indicating a relatively narrow primary size distribution (Figure 1B). Dynamic light scattering analysis revealed a Z-average hydrodynamic diameter of about 210 nm with a polydispersity index (PDI) of 0.101, consistent with a fairly homogeneous colloidal dispersion (Figure 1C). The larger particle size observed by DLS compared with TEM is expected because DLS measures the hydrodynamic diameter of hydrated nanoparticles in suspension, whereas TEM reflects the dry particle size. The PNL-PLGA nanoparticles carried a negative surface charge (zeta potential of −33.9 mV), indicative of good colloidal stability (Figure 1D). The formulation exhibited an entrapment efficiency of 87.02%, indicating efficient incorporation of paeonol into the PLGA matrix.

### 2.2. FTIR Analysis of PNL-PLGA Nanoparticles and Components

**Figure 2:**
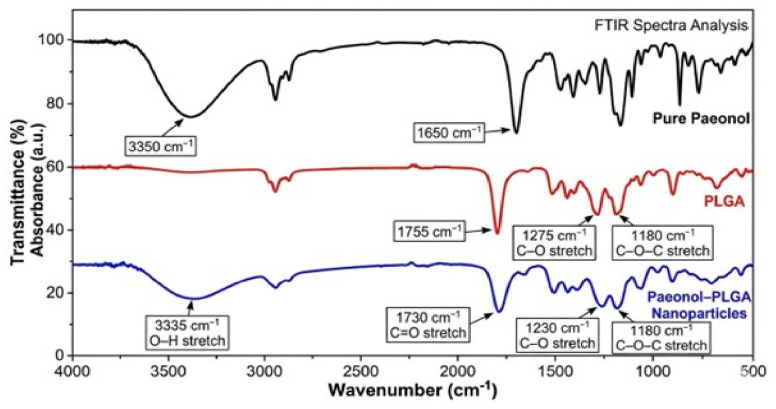
FTIR spectra of paeonol, PLGA, and paeonol-loaded PLGA (PNL-PLGA) nanoparticles, highlighting characteristic functional groups and confirming drug encapsulation.

Fourier transform infrared (FTIR) spectra of pure paeonol, PLGA, and PNL-PLGA nanoparticles are shown in Figure 2. Pure paeonol displayed a broad O–H stretching band around 3350 cm^−1^ and a characteristic C=O stretching peak at approximately 1650 cm^−1^. The PLGA polymer presented a prominent ester C=O stretching band near 1755 cm^−1^, together with bands at about 1275 cm^−1^ and 1180 cm^−1^ attributed to C–O and C–O–C stretching vibrations, respectively. The PNL-PLGA nanoparticles exhibited peaks at around 3335 cm^−1^ (O–H stretch), 1730 cm^−1^ (C=O stretch), 1230 cm^−1^ (C–O stretch), and 1180 cm^−1^ (C–O–C stretch). The presence and slight shifting of these characteristic bands support successful encapsulation of paeonol within the PLGA matrix and possible intermolecular interactions between the drug and the polymer.

### 2.3. In Vitro Release Profile of Paeonol from PLGA Nanoparticles

**Figure 3:**
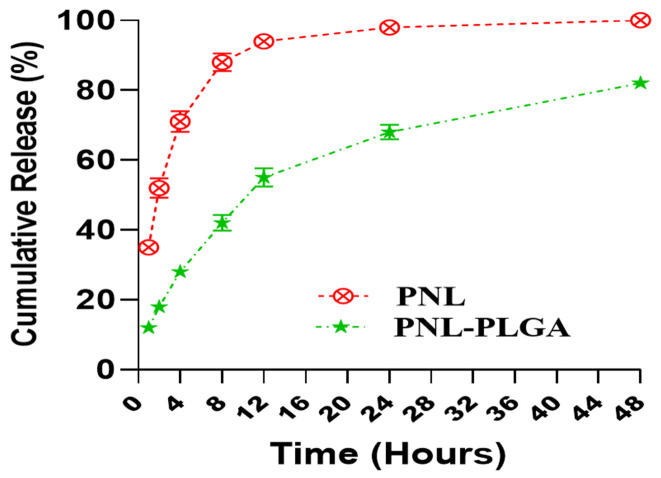
Cumulative release of paeonol (PNL) from free drug solution and paeonol-loaded PLGA (PNL-PLGA) nanoparticles in PBS (pH 7.4) at 37 °C under continuous stirring up to 48 h.

The in vitro release study in phosphate-buffered saline (PBS, pH 7.4) at 37 °C revealed distinct profiles for free paeonol (PNL) and PNL-PLGA nanoparticles (Figure 3). For the free PNL group, paeonol was dispersed in PBS containing a minimal amount of ethanol as a cosolvent to enhance solubility. Free PNL showed a rapid release pattern, with approximately 71% of the drug released within 4 h and almost complete release (about 98%) at 24 h. In contrast, PNL-PLGA exhibited sustained release. Only about 12% of paeonol was released after 1 h, increasing gradually to approximately 42% at 8 h and 68% at 24 h. A slower, continued release was observed thereafter, reaching around 82% by 48 h. These data indicate that PLGA encapsulation effectively retards paeonol release and provides a more controlled release profile compared with the free drug.

### 2.4. Effect of PNL-PLGA Nanoparticles on Rats’ Body Weight and Serum Tumor Markers in DMH-Induced Carcinogenesis

Body weight data are presented in Table 1. DMH-treated rats showed significantly reduced body weight gain compared with controls, whereas co-treatment with free PNL or PNL-PLGA improved weight gain, with greater improvement observed in the PNL-PLGA group.

Serum tumor marker levels are summarized in Table 2. Rats treated with 1,2-dimethylhydrazine (DMH) showed a marked increase in alpha-fetoprotein (AFP), carcinoembryonic antigen (CEA), carbohydrate antigen 19-9 (CA19-9), cancer antigen 125 (CA125), and cancer antigen 15-3 (CA15-3) compared with the control group (*p* < 0.0001 for all), consistent with the induction of a carcinogenic state. Treatment with free paeonol (DMH + PNL) significantly reduced the levels of all measured markers relative to the DMH group, although they remained higher than in the control group in most cases. Co-treatment with PNL-PLGA (DMH + PNL-PLGA) produced a more pronounced attenuation, with CEA and CA19-9 levels in particular approaching levels not significantly different from those of the negative control group.

### 2.5. Effect of PNL-PLGA Nanoparticles on NRF2/HO-1 Pathway and Oxidative Stress in DMH-Induced Carcinogenesis

**Figure 4:**
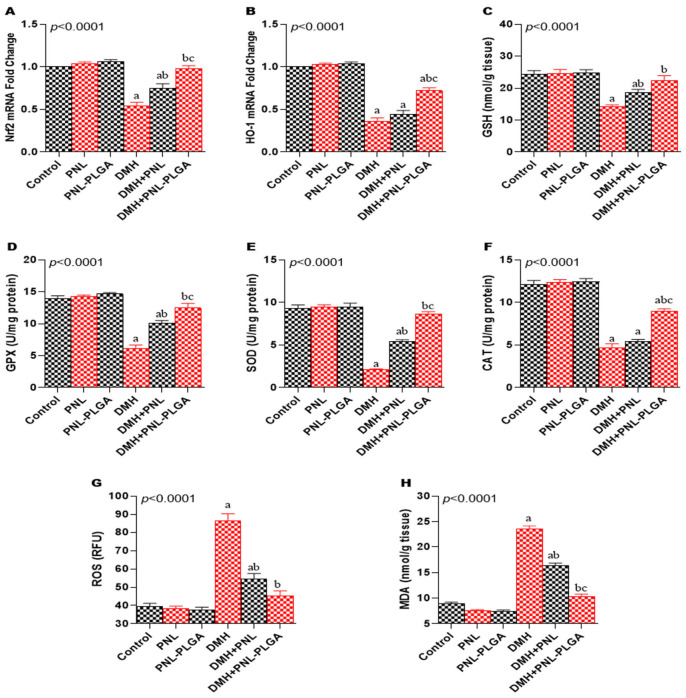
Effect of paeonol (PNL) and paeonol-loaded PLGA (PNL-PLGA) on the NRF2/HO-1 pathway and oxidative stress markers in colonic tissues of DMH-treated rats. (**A**) NRF2 mRNA; (**B**) HO-1 mRNA; (**C**) GSH; (**D**) GPx; (**E**) SOD; (**F**) CAT; (**G**) ROS; (**H**) MDA. Bars with different superscripts (a, b, c) differ significantly at *p* < 0.05 versus control (a), DMH (b), and PNL (c) groups.

Exposure to DMH significantly suppressed the nuclear factor erythroid 2-related factor 2 (NRF2)/heme oxygenase-1 (HO-1) axis compared with the control group, as reflected by reduced mRNA expression of both NRF2 (Figure 4A) and HO-1 (Figure 4B). Co-administration of PNL-PLGA with DMH significantly increased NRF2 and HO-1 expression relative to the DMH group, restoring NRF2 to levels comparable to those of controls. The nanoformulation showed a more pronounced effect than free PNL, particularly for HO-1, where no significant difference was detected between the DMH and DMH + PNL groups.

Antioxidant defenses were also compromised in DMH-treated rats, as evidenced by a significant decrease in colonic reduced glutathione (GSH) content (Figure 4C) and reduced activities of glutathione peroxidase (GPx), superoxide dismutase (SOD), and catalase (CAT) (Figure 4D–F) relative to controls. Co-treatment with either PNL or PNL-PLGA significantly improved GSH levels and GPx and SOD activities, generally restoring them to values not significantly different from those of the control group. CAT activity was significantly increased only in the DMH + PNL-PLGA group, whereas the DMH + PNL group did not differ significantly from the DMH group. Overall, PNL-PLGA produced greater improvements in antioxidant enzyme activities than free PNL.

Consistent with these findings, DMH exposure markedly elevated colonic reactive oxygen species (ROS) (Figure 4G) and malondialdehyde (MDA) levels (Figure 4H) compared with controls. Both PNL and PNL-PLGA co-treatments significantly reduced ROS and MDA; importantly, ROS and MDA levels in the DMH + PNL-PLGA group did not differ significantly from those in the control group. The nanoformulation was therefore more effective than crude PNL in limiting lipid peroxidation and ROS accumulation.

### 2.6. Effect of PNL-PLGA Nanoparticles on DNA Damage Markers in DMH-Induced Carcinogenesis

**Figure 5:**
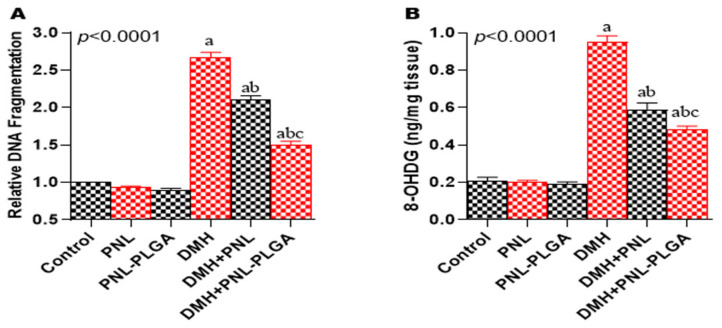
Effect of paeonol (PNL) and paeonol-loaded PLGA (PNL-PLGA) on DNA damage markers in colonic tissues of DMH-treated rats. (**A**) DNA fragmentation; (**B**) 8-OHdG. Bars with different superscripts (a, b, c) differ significantly at *p* < 0.05 versus control (a), DMH (b), and PNL (c) groups.

Markers of DNA damage are presented in Figure 5. DMH administration significantly increased colonic DNA fragmentation (Figure 5A) and levels of 8-hydroxy-2′-deoxyguanosine (8-OHdG; Figure 5B) compared with the control group, indicating enhanced apoptotic fragmentation and oxidative DNA damage. Co-treatment with either PNL or PNL-PLGA significantly reduced both DNA fragmentation and 8-OHdG levels relative to the DMH group. The reduction was partial in the DMH + PNL group, whereas the DMH + PNL-PLGA group showed the most marked decrease, approaching values closer to those observed in control animals.

### 2.7. Effect of PNL-PLGA on Inflammatory Response in DMH-Induced Carcinogenesis

**Figure 6:**
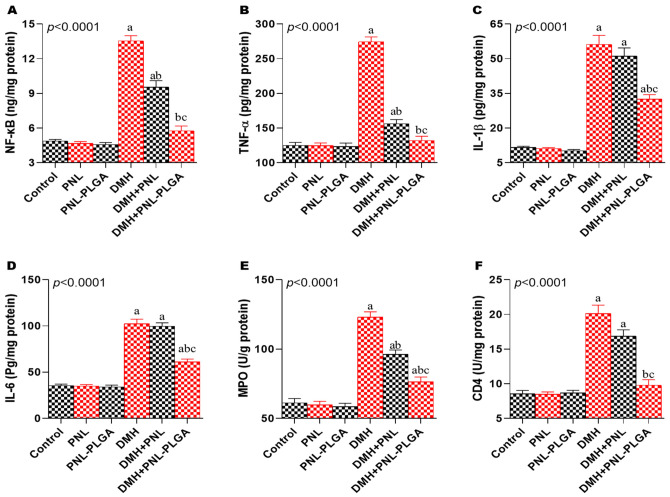
Effect of paeonol (PNL) and paeonol-loaded PLGA (PNL-PLGA) on inflammatory markers in colonic tissues of DMH-treated rats. (**A**) NF-κB; (**B**) TNF-α; (**C**) IL-1β; (**D**) IL-6; (**E**) MPO; (**F**) CD4. Bars with different superscripts (a, b, c) differ significantly at *p* < 0.05 versus control (a), DMH (b), and PNL (c) groups.

Regarding the inflammatory response, 1,2-dimethylhydrazine (DMH) administration led to a significant increase in colonic nuclear factor κB (NF-κB) levels compared with the control group (Figure 6A). Co-treatment with either paeonol (PNL) or paeonol-loaded poly(lactic-co-glycolic acid) nanoparticles (PNL-PLGA) significantly reduced NF-κB, and in the DMH + PNL-PLGA group, NF-κB levels were restored to values that did not differ significantly from those of the control group. Similarly, the pro-inflammatory cytokines tumor necrosis factor-α (TNF-α), interleukin-1β (IL-1β), and interleukin-6 (IL-6) were markedly elevated in the DMH group relative to controls (Figure 6B–D). PNL-PLGA co-treatment significantly decreased all three cytokines, with TNF-α returning to levels comparable to those in the control group. In contrast, crude PNL co-treatment did not significantly alter IL-1β and IL-6 levels compared with the DMH-only group.

Colonic myeloperoxidase (MPO), a marker of neutrophil-associated inflammation, was also significantly higher in DMH-treated rats than in controls (Figure 6E). Both PNL and PNL-PLGA co-treatments significantly reduced MPO, with the greatest reduction observed in the DMH + PNL-PLGA group. In addition, DMH exposure significantly increased colonic cluster of differentiation 4 (CD4) levels compared with the control group (Figure 6F). Co-treatment with crude PNL produced only a partial decrease, whereas PNL-PLGA co-treatment reduced CD4 levels to values not significantly different from those of the control group. Overall, PNL-PLGA showed a better modulatory effect on the inflammatory response than free PNL.

### 2.8. Effect of PNL-PLGA on Autophagy-Related Gene Expression in DMH-Induced Carcinogenesis

**Figure 7:**
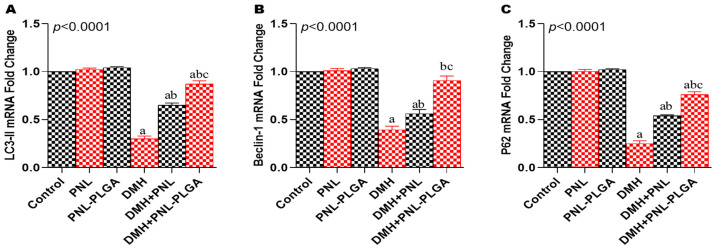
Effect of paeonol (PNL) and paeonol-loaded PLGA (PNL-PLGA) on autophagy-related markers in colonic tissues of DMH-treated rats. (**A**) Beclin-1; (**B**) LC3; (**C**) p62. Bars with different superscripts (a, b, c) differ significantly at *p* < 0.05 versus control (a), DMH (b), and PNL (c) groups.

To assess the impact on autophagy, we measured mRNA expression of Beclin-1, microtubule-associated protein 1 light chain 3 (LC3), and sequestosome-1 (p62) in colonic tissue. DMH exposure significantly downregulated Beclin-1, LC3, and p62 expression compared with the control group (Figure 7A–C), indicating suppression of autophagy-related signaling. Co-treatment with either PNL or PNL-PLGA significantly upregulated the expression of all three genes relative to the DMH group. The most pronounced effect was observed in the DMH + PNL-PLGA group, in which Beclin-1 expression was restored to levels that did not differ significantly from those of controls, while LC3 and p62 were also markedly increased.

### 2.9. Effect of PNL-PLGA on Apoptosis Markers in DMH-Induced Carcinogenesis

**Figure 8:**
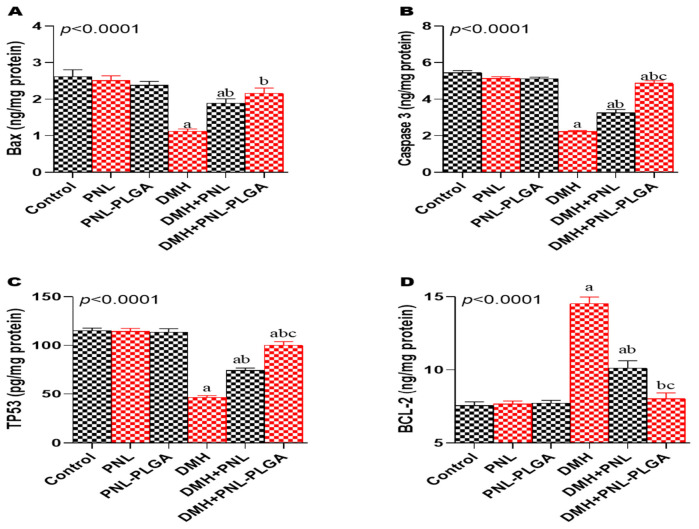
Effect of paeonol (PNL) and paeonol-loaded PLGA (PNL-PLGA) on apoptosis markers in colonic tissues of DMH-treated rats. (**A**) Bax; (**B**) caspase-3; (**C**) TP53; (**D**) Bcl-2. Bars with different superscripts (a, b, c) differ significantly at *p* < 0.05 versus control (a), DMH (b), and PNL (c) groups.

We next examined key proteins involved in apoptosis. DMH treatment significantly decreased the colonic levels of the pro-apoptotic markers Bcl-2-associated X protein (Bax), caspase-3, and tumor protein p53 (TP53) compared with the control group (Figure 8A–C). Co-treatment with either PNL or PNL-PLGA significantly increased Bax, caspase-3, and TP53 relative to DMH alone, with the highest levels consistently observed in the DMH + PNL-PLGA group. In this group, Bax levels did not differ significantly from control values, suggesting near-normalization of the pro-apoptotic signal.

Conversely, the antiapoptotic protein B-cell lymphoma-2 (Bcl-2) was significantly elevated in the DMH group compared with controls (Figure 8D). PNL co-treatment partially reduced Bcl-2, whereas PNL-PLGA co-treatment restored Bcl-2 to values that were not significantly different from those of the control group.

### 2.10. Effect of PNL-PLGA on Liver and Kidney Function Markers in DMH-Induced Carcinogenesis

Serum biochemical parameters are presented in Table 3. DMH administration significantly impaired liver function, as reflected by marked increases in alanine aminotransferase (ALT) and aspartate aminotransferase (AST) activities compared with the control group (*p* < 0.0001 for both). Co-treatment with PNL-PLGA significantly lowered ALT and AST relative to the DMH group and restored their values to levels not significantly different from those of controls. Free PNL also reduced ALT and AST compared with DMH alone, but these values remained higher than in the PNL-PLGA group.

Renal function was similarly affected by DMH, with significant elevations in serum creatinine and urea concentrations compared with control (Table 3). Co-treatment with PNL-PLGA significantly reduced both creatinine and urea relative to DMH alone and showed a greater improvement than crude PNL, particularly for urea, where the DMH + PNL group did not differ significantly from the DMH group.

### 2.11. Histopathological Evaluation of Colonic Tissue

**Figure 9:**
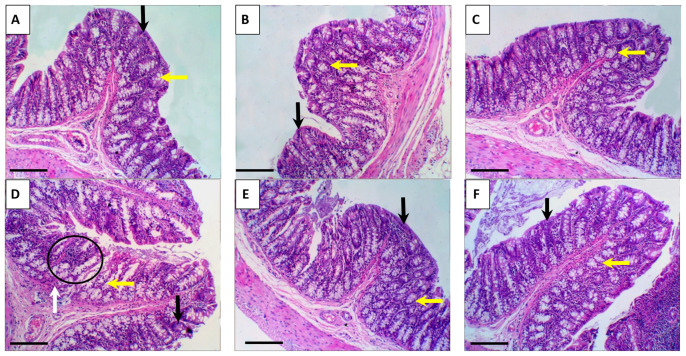
Representative photomicrographs of colonic tissue from the different experimental groups (H&E, 400×; scale bar = 50 µm). (**A**) Control group showing normal colonic mucosa with intact surface epithelium and well-organized tubular crypts (black arrow), and abundant goblet cells within the crypts and surface epithelium (yellow arrow). (**B**) PNL group and (**C**) PNL-PLGA group displaying mucosal architecture comparable to control, with preserved crypt morphology and goblet cell distribution (yellow arrows) and absence of dysplastic changes; the black arrows indicate the intact epithelial surface. (**D**) DMH group exhibiting marked distortion of colonic architecture with irregular, fused, and branching crypts; the black circle highlights areas of complex glandular crowding and epithelial dysplasia, the yellow arrow points to goblet cell depletion, and the white arrow indicates dense inflammatory cell infiltration in the lamina propria; a black arrow marks focal mucosal ulceration/loss of surface epithelium. (**E**) DMH + PNL group showing partial improvement, with better preservation of crypt organization and surface epithelium (black arrow) and reduced, though still evident, dysplastic changes and goblet cell loss (yellow arrow) compared with DMH alone. (**F**) DMH + PNL-PLGA group demonstrating near-normal mucosal architecture, with restoration of crypt arrangement and surface epithelium (black arrow), reappearance of goblet cells (yellow arrow), and minimal inflammatory infiltrate, indicating a pronounced histological protective effect of the paeonol-loaded PLGA nanoparticles.

Histological examination of H&E-stained colon sections from control rats showed preserved mucosal architecture with an intact epithelial lining, regularly arranged crypts, and abundant goblet cells distributed throughout the mucosa. Epithelial cells maintained normal polarity and nuclear morphology (Figure 9A–C). In the DMH group, severe architectural distortion was evident (Figure 9D). Colonic crypts appeared irregular and frequently fused, and epithelial cells displayed marked atypia, including loss of polarity, hyperchromatic nuclei, and an increased nuclear-to-cytoplasmic ratio. Foci of invasive glandular structures were observed, accompanied by prominent inflammatory cell infiltration within the lamina propria and submucosa.

In the DMH + PNL group, partial histological improvement was noted. Colonic crypts were more regularly arranged, epithelial atypia was less pronounced than in the DMH group, and inflammatory cell infiltration was noticeably reduced (Figure 9E). The DMH + PNL-PLGA group showed the most extensive recovery (Figure 9F). The mucosal structure was largely preserved, with well-organized crypts, more frequent goblet cells, minimal epithelial atypia, and only mild inflammatory infiltration.

### 2.12. Ultrastructural Alterations in Colonic Epithelium

**Figure 10:**
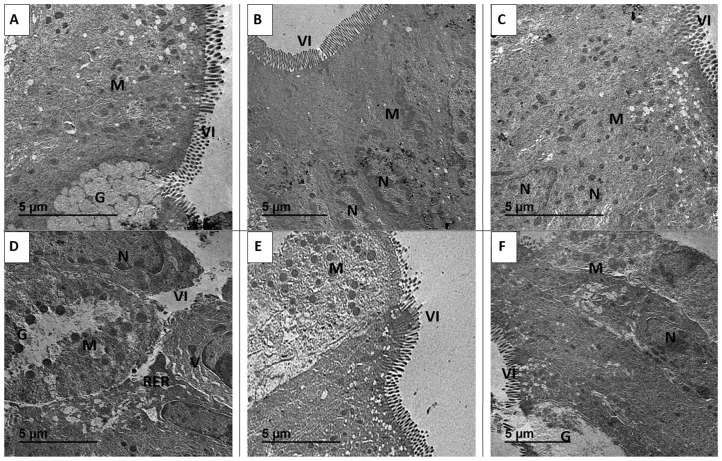
Representative transmission electron microscopy images of colonic epithelium from the different experimental groups (scale bar = 5 µm). (**A**) Control group showing intact epithelial cells with well-defined microvilli (VI), euchromatic nuclei (N), and mitochondria (M), and normal goblet cell secretory granules (G). (**B**) PNL group showing ultrastructure comparable to that of the control, with preserved microvilli and organelles. (**C**) PNL-PLGA group also exhibited normal epithelial ultrastructure, indicating that the nanoformulation alone does not induce subcellular damage. (**D**) DMH group showing pronounced ultrastructural alterations, including irregular or shortened microvilli, swollen and degenerated mitochondria, dilated rough endoplasmic reticulum (RER), nuclear irregularity/condensation, and loss of goblet cell granules, consistent with DMH-induced injury; vacuole (V). (**E**) DMH + PNL group demonstrating partial amelioration, with improvement in microvillar organization and mitochondrial morphology, although some degenerative changes persist. (**F**) DMH + PNL-PLGA group exhibiting near-normal epithelial ultrastructure, with restoration of microvilli, more regular nuclei, and largely preserved mitochondria and goblet cells, supporting the superior protective effect of paeonol-loaded PLGA nanoparticles at the ultrastructural level.

On transmission electron microscopy, colonic epithelial cells from control rats showed preserved ultrastructure. Nuclei were euchromatic and regularly contoured, mitochondria were numerous with intact cristae, and apical microvilli formed a dense, well-organized brush border (Figure 10A–C). In contrast, 1,2-dimethylhydrazine (DMH) treatment induced marked degenerative changes (Figure 10D). Epithelial nuclei appeared irregular with areas of chromatin condensation, and occasional focal disruption of the nuclear envelope was observed. Mitochondria were swollen and exhibited fragmented cristae, while the rough endoplasmic reticulum was markedly dilated and disorganized. The cytoplasm contained prominent electron-lucent vacuoles, and the apical microvilli were reduced in number and irregularly arranged.

In the DMH + paeonol (PNL) group, partial improvement was evident. Nuclear morphology was largely restored, mitochondrial swelling was less prominent than in the DMH group, and apical microvilli appeared more continuous, although not completely normal (Figure 10E). The DMH + paeonol-loaded poly(lactic-co-glycolic acid) (PNL-PLGA) group showed the most preserved ultrastructure (Figure 10F). Colonic epithelial nuclei were mostly regular, mitochondria displayed near-normal morphology with distinct cristae, and the apical microvilli were re-established as a well-aligned brush border, indicating substantial structural recovery.

### 2.13. Effect of PNL Formulations on NRF2 Expression

**Figure 11:**
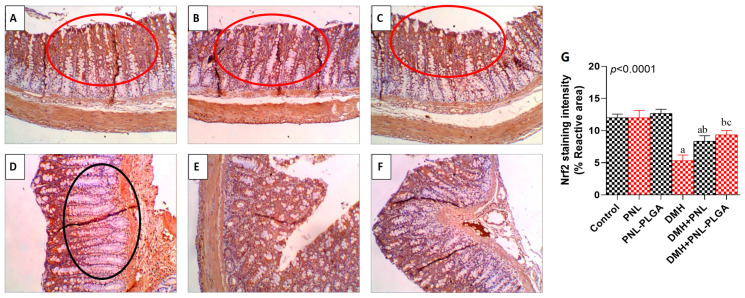
Representative photomicrographs of NRF2 immunostaining in colonic sections. (**A**) Control; (**B**) PNL; (**C**) PNL-PLGA; (**D**) DMH; (**E**) DMH + PNL; (**F**) DMH + PNL-PLGA. Strong nuclear NRF2 immunoreactivity is evident in control and PNL-treated groups, markedly reduced in the DMH group, and partially to markedly restored after PNL or PNL-PLGA co-treatment (×400). (**G**) Semi-quantitative NRF2 immunostaining scores in colonic tissues from the different groups. Bars with different superscripts (a, b, c) differ significantly at *p* < 0.05 versus control (a), DMH (b), and PNL (c) groups.

Immunohistochemical analysis of nuclear factor erythroid 2-related factor 2 (NRF2) in colonic sections revealed marked differences among the groups. In control, PNL, and PNL-PLGA rats, strong NRF2 immunoreactivity was observed in the nuclei of colonic epithelial cells (Figure 11A–C). By contrast, DMH-treated rats showed a pronounced reduction in NRF2 staining, with expression appearing weak to nearly absent in most epithelial cells (Figure 11D).

Co-treatment with free PNL (DMH + PNL) resulted in a clear improvement in NRF2 expression, as reflected by moderately increased immunoreactivity compared with the DMH group (Figure 11E). PNL-PLGA co-treatment (DMH + PNL-PLGA) produced an even more evident increase in NRF2 staining intensity (Figure 11F). Semi-quantitative scoring confirmed a significant decrease in NRF2 in the DMH group relative to controls, and a significant elevation in both treatment groups, with the highest NRF2 scores recorded in the DMH + PNL-PLGA group (Figure 11G).

## 3. Discussion

Colorectal cancer is a major global health burden and one of the leading causes of cancer-related morbidity and mortality worldwide [29]. Its development reflects a complex interplay between environmental and lifestyle factors, including high intake of red and processed meat, low dietary fiber consumption, obesity, physical inactivity, smoking, alcohol use, and chronic inflammatory bowel disease, superimposed on aging and inherited genetic susceptibility [30]. These risk factors converge on molecular pathways that promote genomic instability, chronic inflammation, oxidative stress, and disruption of epithelial homeostasis, ultimately driving the adenoma-carcinoma sequence [31].

In chemically induced models such as the DMH rat model used in this study, these pathogenic processes are recapitulated by sustained generation of reactive metabolites, excessive production of reactive oxygen species, chronic mucosal inflammation, and progressive architectural and cytological atypia in the colonic epithelium [32]. Such models therefore provide a relevant platform for evaluating chemopreventive interventions targeting key hallmarks of colorectal carcinogenesis, including redox imbalance, inflammatory signaling, autophagy, and apoptosis [33].

This study provides a potential mechanistic evaluation of the ameliorative and therapeutic effects of PNL-PLGA nanoparticles in a well-established DMH-induced rat model of colorectal cancer. Colorectal carcinogenesis is a multistep process driven by oxidative stress, chronic inflammation, disrupted apoptosis, genomic instability, and impaired immune surveillance [2,34]. Given this complexity, a broad set of biochemical, molecular, immunological, histopathological, and ultrastructural parameters was assessed. This integrative strategy enabled a more detailed characterization of DMH-induced tumor progression and revealed the ameliorative roles of PNL in both free and nanoencapsulated forms.

DMH-treated rats exhibited a marked increase in serum tumor markers, including CEA, CA19-9, AFP, CA125, and CA15-3, consistent with the development of a malignant state and activation of oncogenic signaling cascades. CEA is closely linked to colorectal cancer progression and contributes to cell adhesion, invasion, and metastasis; elevated levels are associated with more aggressive tumor features and epithelial disruption [35,36]. CA19-9, a mucin-associated glycoprotein, is related to tumor progression and reflects increased secretory activity of transformed epithelial cells [37]. Although AFP, CA125, and CA15-3 are not specific for colorectal cancer, their elevation suggests systemic inflammation, metabolic rearrangements, and tumor-associated signaling across organs [38,39,40]. Treatment with PNL significantly reduced these markers, and the PLGA nanoparticle formulation produced even greater reductions. This likely reflects improved bioavailability and more efficient colonic delivery of PNL, resulting in more effective suppression of tumor progression. These findings are in agreement with previous reports that phenolic compounds can inhibit tumor growth by modulating proliferation pathways, cell adhesion, and cell-cycle progression [41,42].

CEA is associated with colorectal cancer burden, involved in cell adhesion, invasion, and metastatic spread, and its increase reflects epithelial disruption and aggressive tumor. CA19-9, a mucin-associated glycoprotein, is linked to enhanced secretory activity of transformed epithelial cells, while AFP, CA125, and CA15-3, though not specific for colorectal cancer, may indicate systemic inflammatory and metabolic alterations related to neoplastic growth.

Oxidative stress is recognized as a key mechanism in DMH-induced colorectal carcinogenesis [43]. It contributes to early and persistent cell injury and cancer development by disrupting redox homeostasis [44]. Excessive ROS production promotes the oxidation of lipids, proteins, and DNA [45], thereby increasing mutation rates and sustaining tumor development and progression [46]. In the present study, DMH-treated rats showed a clear oxidative imbalance, evidenced by increased ROS and MDA levels and reduced GSH levels and antioxidant enzyme activities (GPx, SOD, and CAT). The marked increase in 8-OHdG confirms oxidative DNA damage, a well-established marker of genomic instability and guanine oxidation [47]. Increased DNA fragmentation further reflects advanced oxidative injury, mutation accumulation, and impaired apoptosis during colorectal cancer development [48,49]. Notably, PNL-PLGA co-treatment reversed these alterations more effectively than free PNL, reducing ROS and lipid peroxidation and restoring antioxidant enzyme activities. These effects point to strong radical-scavenging capacity and reinforcement of the endogenous antioxidant system, in line with earlier findings in cancer models [23,50].

**Figure 12:**
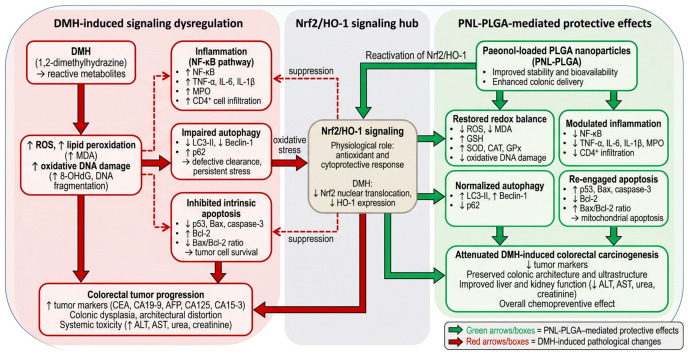
Proposed mechanistic model linking dysregulated signaling pathways in DMH-induced colorectal carcinogenesis and the chemopreventive actions of paeonol-loaded PLGA nanoparticles (PNL-PLGA). DMH exposure generates reactive metabolites that increase ROS and lipid peroxidation, induce oxidative DNA damage, and suppress Nrf2/HO-1 signaling and antioxidant enzymes (SOD, CAT, GPx). This redox imbalance cooperates with activation of NF-κB and pro-inflammatory cytokines (TNF-α, IL-6, IL-1β, MPO), impaired autophagic flux (↓LC3-II, ↓Beclin-1, ↑p62), and inhibition of intrinsic mitochondrial apoptosis (↓p53, Bax, caspase-3; ↑Bcl-2) to promote genomic instability, dysregulated cell survival, and colorectal tumor progression, accompanied by increased tumor markers and systemic organ toxicity. PNL-PLGA enhances colonic delivery of paeonol, leading to reactivation of Nrf2/HO-1-dependent cytoprotective responses, restoration of antioxidant defenses, attenuation of NF-κB-driven inflammation, normalization of autophagy, and re-engagement of apoptosis, thereby preserving colonic architecture and ultrastructure and mitigating DMH-induced colorectal carcinogenesis. Adapted from the open source GAABSTRACT (https://gaabstract.com/) (accessed on 19 June 2026).

At the signaling level, DMH exposure inhibited the Nrf2/HO-1 pathway, indicating impaired cellular protection against oxidative stress. Nuclear factor erythroid 2-related factor 2 (Nrf2) regulates a battery of antioxidant and detoxifying genes and controls enzymes such as heme oxygenase-1 (HO-1), which contribute to redox balance and cytoprotection [51,52]. In normal and premalignant tissue, appropriate activation of Nrf2/HO-1 limits oxidative DNA damage, maintains epithelial barrier integrity, and restrains inflammation, thereby exerting chemopreventive effects [53]. However, sustained or constitutive Nrf2 activation in established tumors has also been implicated in enhanced survival, metabolic reprogramming, and therapy resistance, highlighting a context-dependent “double-edged sword” role for this pathway in cancer [54,55]. In the present DMH model, the predominant finding was suppression of Nrf2/HO-1 signaling, consistent with loss of an important early defense mechanism during carcinogenesis. Conversely, PNL administration, particularly in nanoparticle form, significantly activated the Nrf2/HO-1 pathway. This activation may enhance antioxidant defenses, facilitate detoxification of reactive intermediates, and reduce oxidative DNA injury. These findings support the notion that PNL acts not only as a direct antioxidant but also indirectly by upregulating endogenous cytoprotective signaling pathways, suggesting that the nanoformulation restores a physiological, rather than excessive, level of Nrf2/HO-1 activity in DMH-damaged colonic epithelium [11]. Taken together, these observations position Nrf2/HO-1 as a central hub connecting redox balance, inflammatory responses, autophagy, and apoptosis in this DMH model, and provide the basis for the integrated mechanistic scheme presented in Figure 12.

Chronic inflammation is another major driver of colorectal cancer development. In this study, DMH treatment significantly increased colonic levels of NF-κB, TNF-α, IL-6, IL-1β, and MPO, indicating sustained inflammatory activity. NF-κB is a pivotal transcription factor linking inflammation and cancer by regulating genes involved in tumor growth, metastasis, survival, and angiogenesis [56]. Pro-inflammatory cytokines such as TNF-α, IL-6, and IL-1β maintain tumor-promoting inflammation and can suppress apoptosis [57], whereas elevated MPO reflects neutrophil-driven oxidative damage [58]. PNL administration lowered these inflammatory mediators, with greater efficacy observed for the nanoparticle formulation, suggesting more effective disruption of inflammation-driven tumor signaling. This is consistent with previous reports indicating that PNL interrupts inflammatory feedback loops within the tumor microenvironment, thereby reducing protumor signaling [59]. Importantly, NF-κB inhibition is known to decrease cytokine production and facilitate restoration of apoptosis in cancer cells [60], which aligns with the improved apoptotic profile seen in the PNL-PLGA-treated group.

DMH exposure was also associated with a significant increase in colonic CD4 protein levels, indicative of enhanced immune cell infiltration and activation within the tumor microenvironment [17]. This elevation reflects a chronic inflammatory state and a dysregulated adaptive immune response in the colon [61]. Persistent inflammatory stress in DMH-induced carcinogenesis contributes to immune imbalance and altered T-cell regulation in colonic tissue. PNL-PLGA administration reduced CD4 levels more effectively than crude PNL, suggesting a stronger anti-inflammatory and immunomodulatory effect at the tissue level. The normalization of CD4 expression points to partial restoration of immune homeostasis and a more regulated microenvironment that may support antitumor activity [62].

Autophagy is an essential cellular maintenance mechanism that degrades and recycles damaged organelles and macromolecules. In the context of cancer, its suppression favors accumulation of defective organelles, persistent oxidative stress, and genomic instability, thereby supporting tumor development and progression [44,63]. In this study, DMH exposure significantly reduced LC3-II and Beclin-1 expression, while p62 expression was markedly increased, indicating impaired autophagic flux and suppression of autophagy. This impairment may exacerbate oxidative stress, compromise cellular clearance mechanisms, and facilitate tumor evolution. PNL-PLGA treatment significantly restored autophagic balance, as reflected by increased LC3-II and Beclin-1 expression together with reduced p62 levels. These results are consistent with those of Gao et al., who reported that PNL promotes autophagy by suppressing Akt/mTOR signaling and activating autophagic flux in cancer cells [64]. Overall, restored autophagy in the PNL-PLGA group is closely linked to the reactivation of apoptosis and may contribute to inhibiting tumor progression.

Apoptosis is critical for eliminating genetically damaged and transformed cells, and its disruption is a hallmark of cancer progression [65]. In the present study, DMH exposure reduced colonic levels of TP53, Bax, and caspase-3 while increasing Bcl-2, indicating suppression of intrinsic mitochondrial apoptosis and promotion of tumor cell survival. PNL-PLGA co-treatment was more effective than crude PNL in normalizing these apoptosis-related markers. This recovery of apoptosis likely results from several interconnected mechanisms. Among them, TP53 reactivation is particularly important, as p53 senses DNA damage and initiates mitochondrial apoptosis [66]. It upregulates pro-apoptotic genes such as Bax and ultimately leads to caspase-3 activation and programmed cell death [67]. At the same time, an increase in the Bax/Bcl-2 ratio favors mitochondrial membrane permeabilization, cytochrome c release, and activation of the caspase cascade, all of which are key events in intrinsic apoptosis [68]. Moreover, improved apoptosis is likely linked to the antioxidant and anti-inflammatory properties of PNL, since high ROS levels and NF-κB activation can suppress p53 function and enhance Bcl-2 expression, thereby supporting tumor cell survival [69].

Beyond tumor development, DMH carcinogenesis was associated with systemic toxicity in major organs, particularly the liver and kidneys [17]. These organs are highly susceptible to oxidative and inflammatory damage because of their central roles in detoxification, metabolism, and excretion [70,71]. Accordingly, liver and kidney biomarkers provide important information on the systemic impact of carcinogenesis and the extent of therapeutic protection. In this study, DMH exposure significantly increased serum ALT, AST, urea, and creatinine levels, indicating hepatic and renal dysfunction. These changes are consistent with systemic toxicity driven by oxidative stress, inflammation, and metabolic dysregulation during tumor progression [17]. Elevations in ALT and AST reflect hepatic injury and membrane leakage, often attributable to ROS-induced lipid peroxidation and mitochondrial dysfunction [72]. Similarly, increased urea and creatinine suggest reduced renal filtration and nephron damage, likely due to oxidative stress and inflammatory infiltration in kidney tissue [73]. PNL-PLGA administration markedly reversed these alterations, indicating substantial ameliorative effect against DMH-induced hepatic and renal toxicity and supporting the nanoformulation’s systemic safety profile.

Finally, histological and ultrastructural observations corroborated the biochemical and molecular findings. In DMH-treated rats, colonic tissues exhibited severe disruption of normal architecture, including loss of crypt organization, epithelial hyperplasia, goblet cell depletion, and dense inflammatory cell infiltration, together with evident dysplastic changes. Ultrastructurally, electron microscopy revealed pronounced cellular injury, such as mitochondrial swelling with disrupted cristae, nuclear abnormalities, membrane damage, and cytoplasmic vacuolization. These alterations indicate profound subcellular oxidative and metabolic injury and align with the observed increases in oxidative stress, inflammation, and dysregulated autophagy and apoptosis. PNL treatment alleviated many of these abnormalities, with more pronounced improvement in the PNL-PLGA group. Colonic tissues in PNL-PLGA-treated rats showed near-normal mucosal architecture, restored crypt organization, reduced inflammatory infiltration, and preserved epithelial integrity. Ultrastructural analysis confirmed better mitochondrial preservation, improved nuclear morphology, and reduced cytoplasmic vacuolization, indicating an ameliorative effect against DMH-induced colonic damage.

Overall, these findings suggest that PNL-PLGA nanoparticles exert multi-level ameliorative effects in DMH-induced colorectal carcinogenesis by modulating dysregulated redox, inflammatory, autophagic, apoptotic, and immune signaling pathways, with reactivation of Nrf2/HO-1-dependent cytoprotective responses emerging as a central mechanism. This concept is summarized in Figure 12, which illustrates how PNL-PLGA-mediated restoration of Nrf2/HO-1 signaling counteracts DMH-induced oxidative stress, dampens NF-κB-driven inflammation, normalizes autophagy, and re-engages mitochondrial apoptosis to limit colorectal tumor development.

This study has some limitations that should be acknowledged. It relies on a single chemically induced model of colorectal carcinogenesis, the DMH rat model, which, despite reproducing many features of human disease, does not fully reflect the genetic diversity, stromal complexity, and long-term course of colorectal cancer in patients. The work also focused on tissue-level modulation of oxidative stress, inflammation, autophagy, apoptosis, and NRF2/HO-1-linked redox signaling, without dissecting upstream oncogenic cascades such as PI3K/AKT/mTOR, Ras/Raf/MEK/ERK, or Wnt/β-catenin in detail, nor differentiating between early chemopreventive and potential late pro-survival roles of Nrf2 in fully established tumors. In addition, pharmacokinetic, biodistribution, and extended toxicological evaluations of paeonol-loaded PLGA nanoparticles were not performed, so the contribution of altered drug exposure and the long-term safety profile of this formulation remain to be clarified. Moreover, long-term clinical outcomes, including survival analysis and tumor progression monitoring, were not evaluated, limiting assessment of whether the observed molecular and histopathological improvements translate into sustained therapeutic benefit. The nanoformulation used is a non-targeted PLGA system, and potential advantages of active targeting strategies were not explored. Finally, the experiments were conducted in male rats over a limited time frame; possible sex-related differences and longer-term outcomes, including metastasis and survival, were beyond the scope of this work and warrant further investigation.

## 4. Materials and Methods

### 4.1. Preparation and Physicochemical Characterization of Paeonol-Loaded PLGA Nanoparticles

Paeonol-loaded PLGA nanoparticles (PNL-PLGA) were prepared according to the method of Mehrotra and Pandit [74] with minor modifications. Briefly, 100 mg paeonol and 200 mg PLGA were dissolved in 3 mL of acetone to obtain the organic phase. In parallel, a 1–2% (*w*/*v*) polyvinyl alcohol (PVA) solution was prepared as the aqueous stabilizing phase and kept on ice. The organic solution was slowly added to the chilled PVA under continuous magnetic stirring, followed by high-speed homogenization at 15,000 rpm for 5 min (Ultra-Turrax T25, IKA, Staufen, Germany), yielding a coarse emulsion. This pre-emulsion was further processed using probe sonication (SONOPULS HD 3200, Bandelin electronic GmbH & Co. KG, Berlin, Germany) at 80% amplitude with 10-s intermittent pulses to obtain a nanosuspension of PNL-PLGA. The nanosuspension was magnetically stirred at room temperature for 3 h to ensure complete evaporation of the organic solvent and stabilization of the nanoparticles. The final suspension was collected and stored at 4 °C until use.

The hydrodynamic diameter, polydispersity index (PDI), and zeta potential of PNL-PLGA were determined by dynamic light scattering using a Zetasizer Nano ZS (Malvern Panalytical Ltd., Malvern, UK) after appropriate dilution. Measurements were performed in triplicate. Particle morphology was examined by transmission electron microscopy (TEM; JEM-2100, JEOL Ltd., Tokyo, Japan) operated at 160 kV. Encapsulation efficiency (EE%) was calculated as:

EE (%) = [(initial paeonol amount − unencapsulated paeonol)/initial paeonol amount] × 100 [(initial paeonol amount−unencapsulated paeonol)/initial paeonol amount] × 100.

### 4.2. FTIR Analysis

Fourier transform infrared (FTIR) spectroscopy was employed to assess potential interactions between paeonol and the polymer matrix. Dried samples of pure paeonol, PLGA, and lyophilized PNL-PLGA were mixed with KBr, pressed into pellets, and scanned in the 4000–400 cm^−1^ range. Shifts or changes in characteristic peaks were evaluated to confirm successful encapsulation and polymer–drug interactions.

### 4.3. In Vitro Drug Release

The in vitro release of paeonol from PNL-PLGA was evaluated in phosphate-buffered saline (PBS, pH 7.4) at 37 °C. A known volume of nanoparticle suspension was placed in a dialysis bag and immersed in PBS under gentle stirring. At predefined time points, aliquots were withdrawn from the release medium and replaced with fresh PBS to maintain sink conditions. Paeonol concentration in the samples was determined spectrophotometrically, and cumulative release was calculated over time.

### 4.4. Animals and Housing

Sixty healthy male Wistar rats (220–250 g) were obtained from the MERC Center, Faculty of Medicine, Mansoura University, Egypt. Animals were housed in clean, well-ventilated cages under controlled environmental conditions (relative humidity 50 ± 6.19%, temperature 25 ± 2.13 °C, 12-h light/dark cycle) and acclimatized for two weeks before experimentation. Rats had ad libitum access to a standard pellet diet and water. Health status was monitored regularly. All animal procedures complied with institutional ethical guidelines and the ARRIVE recommendations for reporting animal research [75]. The study was approved by the Animal Care Unit Committee, Faculty of Veterinary Medicine, Mansoura University, Egypt, registration code number MU-ACUC; VM.R.26.05.289.

### 4.5. In Vivo Experimental Design and Treatments

Rats were randomly assigned to six groups (n = 10 each). Group 1 (control) received 30% ethanol (vehicle) by oral gavage once daily for 10 weeks. Group 2 received free paeonol (PNL, 60 mg/kg) in 30% ethanol orally once daily for 10 weeks, based on an established safe dose [76]. Group 3 received PNL-PLGA at an equivalent paeonol dose (60 mg/kg) once daily for 10 weeks. The same nominal dose was used for both free PNL and PNL-PLGA to enable direct comparison of nanoencapsulation effects while maintaining equivalent paeonol exposure across treatment groups.

Group 4 (DMH) received 1,2-dimethylhydrazine (DMH, 20 mg/kg in 0.9% saline) orally once weekly for 10 weeks [77], along with a daily vehicle (30% ethanol). Group 5 received DMH as in Group 4, plus free PNL (60 mg/kg/day). Group 6 received DMH as in Group 4 plus PNL-PLGA (60 mg/kg/day). All formulations were freshly prepared; PNL-PLGA was suspended in 30% ethanol to ensure dose uniformity. Body weight was recorded weekly to adjust DMH dosing.

### 4.6. Tissue Collection and Processing

At the end of the experiment, rats were fasted for 10 h before euthanasia. Anesthesia was induced with isoflurane, and loss of reflexes was confirmed. Euthanasia was completed by exposure to 5% isoflurane in oxygen for 5 min, and the absence of cardiac and respiratory activity verified death. For biochemical and molecular analyses, 7 samples per group were used; 3 per group were reserved for detailed histopathological and ultrastructural evaluation.

Blood was collected in plain tubes, allowed to clot, and centrifuged at 3000 rpm for 10 min at 4 °C. Serum was aliquoted and stored at −80 °C. Colonic segments were excised, rinsed in saline to remove luminal contents, and divided. One portion was wrapped in aluminum foil and stored at −80 °C for biochemical assays; the other was fixed in 10% neutral buffered formalin for histology, ultrastructure, and immunohistochemistry. For biochemical assays, colon tissue was homogenized in ice-cold phosphate buffer (50 mM, pH 7.4) to obtain 10% (*w*/*v*) homogenates, then centrifuged at 2600× *g* for 20 min. The supernatant was collected and stored at −80 °C.

### 4.7. Serum Biochemistry and Tumor Markers

Serum alanine aminotransferase (ALT), aspartate aminotransferase (AST), urea, and creatinine were determined using validated colorimetric or ELISA-based commercial kits (Supplementary Table S1), following the manufacturers’ protocols. CEA, CA19-9, CA-125, CA15-3, and AFP were quantified using rat-specific sandwich ELISA kits (Supplementary Table S1). Absorbance was read with a microplate reader, and concentrations were calculated from standard curves.

### 4.8. Colonic Redox Status and Oxidative Stress Markers

Colonic redox homeostasis was assessed by measuring reduced glutathione (GSH) and the activities of superoxide dismutase (SOD), catalase (CAT), and glutathione peroxidase (GPx) using commercial colorimetric kits (Supplementary Table S1) according to the manufacturers’ instructions. Lipid peroxidation was evaluated by determining malondialdehyde (MDA) using the thiobarbituric acid-reactive substances (TBARS) method. Spectrophotometric readings were taken at the appropriate wavelengths, and results were expressed as specified by each kit. Intracellular ROS production in colonic cells was determined fluorometrically using a DCFH-DA-based assay (Supplementary Table S1). Freshly isolated colonic cells were incubated with the probe, and fluorescence intensity (excitation at 488 nm, emission at 525 nm) was recorded and normalized to the control.

### 4.9. Oxidative DNA Damage and Apoptotic DNA Fragmentation

Oxidative DNA damage was quantified by measuring 8-hydroxy-2′-deoxyguanosine (8-OHdG) in colon tissue homogenates using a competitive ELISA kit (Supplementary Table S1). Apoptosis-related DNA fragmentation was evaluated using a sandwich ELISA kit (Supplementary Table S1). Assays were performed according to the manufacturer’s instructions, and absorbance was measured with a microplate reader.

### 4.10. Inflammatory Biomarkers, MPO Activity, and CD4

Colonic tumor necrosis factor-α (TNF-α), interleukin-1β (IL-1β), interleukin-6 (IL-6), and nuclear factor-κB (NF-κB) were measured using commercial ELISA kits (Supplementary Table S1). Results were normalized to total protein and run in triplicate. Myeloperoxidase (MPO) activity was assessed spectrophotometrically according to Haqqani et al. [78], based on the H_2_O_2_-dependent oxidation of o-dianisidine. Absorbance was recorded at 460 nm, and activity was expressed as units per gram tissue using the appropriate molar extinction coefficient. CD4 levels in colonic tissue were determined using a quantitative sandwich ELISA kit (Supplementary Table S1).

### 4.11. Apoptosis-Related Proteins and p53

Protein levels of Bax, Bcl-2, and caspase-3 in colon tissue were quantified using specific ELISA kits (Supplementary Table S1). Tumor protein p53 (TP53) was measured with a rat-specific ELISA kit (Supplementary Table S1). All assays were performed according to the manufacturer’s protocols.

### 4.12. RNA Isolation, cDNA Synthesis, and RT-qPCR

Gene expression profiling was conducted on colonic tissue specimens from seven rats per group (n = 7). Total RNA was extracted from individual samples using QIAzol Lysis Reagent (Qiagen GmbH, Hilden, Germany) following mechanical tissue disruption. Phase separation was achieved by adding chloroform and centrifuging at 12,000× *g* for 15 min at 4 °C. The RNA-containing aqueous phase was carefully recovered, and nucleic acids were precipitated with isopropanol, centrifuged (12,000× *g*, 10 min, 4 °C), and washed twice with 75% ethanol to remove residual contaminants. The purified RNA pellet was air-dried and dissolved in DNase/RNase-free water. RNA concentration and purity were assessed spectrophotometrically; samples with A260/A280 ratios between 1.8 and 2.0 were accepted for downstream analysis. RNA integrity was evaluated by agarose gel electrophoresis, confirming the presence of intact 28S and 18S ribosomal RNA bands with a 28S/18S ratio ≥ 1.8, indicating acceptable RNA quality for gene expression analysis. Possible genomic DNA contamination was assessed by including no-reverse-transcriptase (no-RT) controls in each run; no amplification was detected in any no-RT control, confirming the absence of genomic DNA carryover. A fixed input of 500 ng total RNA per sample was used for first-strand cDNA synthesis, performed with the iScript™ cDNA Synthesis Kit (Bio-Rad, Hercules, CA, USA) according to the manufacturer’s protocol. This kit employs a blend of oligo(dT) and random hexamer primers to ensure comprehensive transcriptome coverage and reproducible reverse transcription across all samples. Quantitative PCR amplification was carried out on a Rotor-Gene Q real-time PCR system (Qiagen GmbH, Hilden, Germany) using iTaq™ Universal SYBR^®^ Green Supermix (Bio-Rad). Each reaction was performed in technical duplicate, and no-template controls (NTCs) were included on every plate to monitor reagent contamination; no amplification was observed in any NTC. Amplification efficiency for each primer pair was determined from a five-point, four-fold serial dilution standard curve prepared from a pooled cDNA sample representing all experimental groups. All assays demonstrated amplification efficiencies of 90–105%, with correlation coefficients (R^2^) ≥ 0.98, confirming adequate quantification across the tested dynamic range. Individual primer efficiencies were as follows: Nrf2, 94%; HO-1, 96%; p62, 93%; LC3, 95%; Beclin-1, 92%; β-actin, 97%. Primer sequences, amplicon lengths, and GenBank accession numbers are provided in Table 4. Melt curve analysis was performed at the end of each amplification run to confirm single-product amplification and the absence of primer dimers or non-specific products; a single sharp dissociation peak was observed for all assays.

Beta-actin (β-actin) was selected as the reference gene for normalization based on its reported stability in colonic tissue under oxidative and inflammatory conditions in comparable rat models. Its expression stability across the six experimental groups was confirmed by calculating the coefficient of variation (CV) of raw Cq values, which did not exceed 1.5 cycles across all samples, consistent with acceptable reference gene stability under the experimental conditions used. Relative mRNA expression levels were quantified using the 2^−ΔΔCt^ method [79]. The assumption of comparable amplification efficiencies between target and reference genes required by this method was verified by the efficiency data described above, with all target gene efficiencies within ±5% of β-actin. Results are expressed as fold change relative to the control group mean. All procedures were in accordance with the updated minimum information for publication of quantitative real-time PCR experiments (MIQE 2.0) guidelines [80].

### 4.13. Histology

Colon samples were fixed in 10% neutral buffered formalin for at least 72 h, dehydrated through graded ethanol, cleared in xylene, and embedded in paraffin. Sections (4–5 µm) were cut, deparaffinized, rehydrated, and stained with hematoxylin and eosin (H&E). Stained sections were dehydrated, cleared, mounted, and examined under a light microscope.

### 4.14. Transmission Electron Microscopy

Ultrastructural evaluation of colonic tissue was carried out using TEM. Tissue fragments were promptly trimmed and submerged in a solution of 2.5% glutaraldehyde buffered with 0.1 M phosphate buffer (pH 7.4) at 4 °C for 24 h to achieve optimal primary fixation of subcellular constituents. Specimens were then rinsed thoroughly in the same buffer and transferred to 1% osmium tetroxide for 2 h to accomplish secondary fixation and confer electron opacity to membranous structures. A stepwise ethanol dehydration protocol was applied, followed by acetone clearance, before the tissue was fully infiltrated and embedded in epoxy resin. Using an ultramicrotome, sections 60–70 nm thick were cut and collected on copper support grids. Contrast enhancement was achieved by sequential incubation with uranyl acetate and lead citrate solutions. All grids were visualized under a JEOL 2100 TEM (JEOL Ltd., Tokyo, Japan) at an accelerating voltage of 160 kV.

### 4.15. Immunohistochemistry for NRF2

Paraffin-embedded colon sections were deparaffinized, rehydrated, and subjected to antigen retrieval in 0.01 M citrate buffer (pH 6.0). Endogenous peroxidase activity was blocked with 3% H_2_O_2_ in methanol. After washing in PBS, sections were blocked with 5% bovine serum albumin and incubated overnight at 4 °C with an anti-NRF2 primary antibody (1:200, Abcam, ab62352, Cambridge, UK). After PBS washing, sections were incubated with biotinylated goat anti-rabbit secondary antibody, followed by streptavidin-HRP. Immunoreactivity was visualized with 3,3′-diaminobenzidine (DAB) and counterstained with Mayer’s hematoxylin. Slides were dehydrated, cleared, mounted, and examined at ×400 magnification. NRF2 immunostaining was quantified by an investigator blinded to group allocation using ImageJ software (version 1.54; National Institutes of Health, Bethesda, MD, USA) Five randomly selected fields per section were analyzed, and the percentage of positively stained area and mean optical density were quantified after applying a consistent threshold setting to all images.

### 4.16. Statistical Analysis

Statistical analyses were performed using SAS software (Proc ANOVA; SAS Institute, Cary, NC, USA; version 9.4, 2012). Before applying parametric tests, data were screened for normality using the Shapiro–Wilk criterion and for equality of variances using Levene’s test; both assumptions were satisfied across all datasets. Where statistically significant differences were identified by one-way ANOVA, post hoc pairwise comparisons were conducted using Tukey’s test. Throughout the manuscript, continuous variables are reported as mean ± SE, and differences are considered statistically significant at *p* < 0.05. Data were organized in Microsoft Excel, and all graphical representations were prepared with GraphPad Prism v9.0 (GraphPad Software, Boston, MA, USA).

## 5. Conclusions

In this DMH-induced rat model of colorectal carcinogenesis, DMH caused severe colonic injury characterized by oxidative stress, inflammation, DNA damage, dysregulated apoptosis and autophagy, hepatic and renal dysfunction, and marked histological and ultrastructural alterations. Treatment with free paeonol partially ameliorated these changes, whereas paeonol-loaded PLGA nanoparticles produced more pronounced improvements, including restoration of antioxidant defenses, reduction in inflammatory and oxidative markers, normalization of NRF2/HO-1, autophagy- and apoptosis-related gene and protein expression, and better preservation of colonic architecture and ultrastructure.

Overall, PNL-PLGA nanoparticles showed superior ameliorative efficacy compared with free paeonol in this experimental setting. These findings support nanoparticle-based delivery as a promising approach to enhance the anticarcinogenic potential of natural compounds in colorectal cancer, primarily through modulation of NRF2/HO-1-linked oxidative, inflammatory, autophagic, and apoptotic signaling, and justify further mechanistic, pharmacokinetic, and translational investigations of paeonol-PLGA in advanced preclinical models.

## Figures and Tables

**Table 1:** Effect of paeonol (PNL) and paeonol-loaded PLGA (PNL-PLGA) nanoparticles on rats’ body weight (BW) in DMH-induced colorectal carcinogenesis in rats.

Group	Initial BW (g)	Final BW (g)	BW Change (g)	% Change
Control	234.6 ± 8.3	312.4 ± 11.6	+77.8 ± 9.4	+33.2
PNL	236.1 ± 7.9	318.7 ± 10.8	+82.6 ± 8.7	+35.0
PNL-PLGA	235.4 ± 8.5	324.1 ± 12.1	+88.7 ± 9.1	+37.7
DMH	233.8 ± 9.1	255.4 ± 11.7 ^a^	+21.6 ± 6.8 ^a^	+9.2
DMH + PNL	234.9 ± 8.7	281.6 ± 12.3 ^b^	+46.7 ± 7.4 ^b^	+19.9
DMH + PNL-PLGA	235.7 ± 7.8	297.8 ± 11.2 ^bc^	+62.1 ± 8.0 ^bc^	+26.4

Data are presented as mean ± standard deviation. BW: body weight; g: grams; %: percentage. Distinct superscripts (a, b, c) denote statistically significant differences (*p* < 0.05) relative to the control (a), DMH (b), and PNL (c) groups.

**Table 2:** Effect of paeonol (PNL) and paeonol-loaded PLGA (PNL-PLGA) nanoparticles on serum tumor markers in DMH-induced colorectal carcinogenesis in rats.

Treatments	AFB (ng/mL)	CEA (ng/mL)	CA19-9 (ng/mL)	CA125 (U/mL)	CA15-3 (U/mL)
Control	2.43 ± 0.35	1.89 ± 0.23	23.21 ± 1.54	9.22 ± 0.71	4.01 ± 0.35
PNL	2.34 ± 0.21	1.86 ± 0.25	24.34 ± 2.12	8.45 ± 0.69	3.83 ± 0.23
PNL-PLGA	2.41 ± 0.29	1.92 ± 0.36	21.32 ± 1.23	8.55 ± 0.83	4.22 ± 0.54
DMH	13.25 ± 0.71 ^a^	7.54 ± 0.66 ^a^	47.19 ± 5.44 ^a^	34.23 ± 4.34 ^a^	11.21 ± 0.60 ^a^
DMH + PNL	8.12 ± 0.56 ^ab^	4.11 ± 0.45 ^ab^	32.12 ± 3.76 ^ab^	19.12 ± 2.44 ^ab^	6.23 ± 0.51 ^ab^
DMH + PNL-PLGA	5.38 ± 0.48 ^abc^	2.45 ± 0.29 ^bc^	25.33 ± 2.50 ^b^	13.28 ± 2.12 ^ab^	5.36 ± 0.43 ^ab^
*p*-values	<0.0001	<0.0001	<0.0001	<0.0001	<0.0001

AFP (Alpha-fetoprotein), CEA (Carcinoembryonic antigen), CA19-9 (Carbohydrate antigen 19-9), CA125 (Cancer antigen 125), and CA15-3 (Cancer antigen 15-3). Distinct superscripts (a, b, c) denote statistically significant differences (*p* < 0.05) relative to the control (a), DMH (b), and PNL (c) groups.

**Table 3:** Effect of paeonol (PNL) and paeonol-loaded PLGA (PNL-PLGA) nanoparticles on liver and kidney function markers in DMH-induced colorectal carcinogenesis in rats.

Treatments	ALT (U/L)	AST (U/L)	CR (mg/dL)	Urea (mg/dL)
Control	61.01 ± 3.21	78.27 ± 3.04	0.83 ± 0.04	33.43 ± 2.65
PNL	59.55 ± 3.65	77.56 ± 3.66	0.81 ± 0.03	31.12 ± 3.66
PNL-PLGA	58.32 ± 2.67	75.43 ± 3.57	0.80 ± 0.06	30.43 ± 3.43
DMH	123.23 ± 6.87 ^a^	133.23 ± 9.87 ^a^	1.61 ± 0.15 ^a^	58.60 ± 5.44 ^a^
DMH + PNL	87.44 ± 5.33 ^ab^	110.21 ± 7.12 ^ab^	1.12 ± 0.12 ^ab^	53.55 ± 4.76 ^a^
DMH + PNL-PLGA	66.03 ± 4.01 ^bc^	84.34 ± 6.56 ^bc^	0.98 ± 0.05 ^ab^	45.22 ± 4.77 ^ab^
*p*-values	<0.0001	<0.0001	<0.0001	<0.0001

ALT, alanine aminotransferase; AST, aspartate aminotransferase; CR, creatinine; Urea, blood urea nitrogen. Bars with different superscripts (a, b, c) differ significantly at *p* < 0.05 versus control (a), DMH (b), and PNL (c) groups.

**Table 4:** Primers employed for quantitative real-time PCR analysis of gene expression in rat colonic tissues.

Gene	Sequences (5′-3′)	Accession No	Length (bp)
*Nrf2*	F: TTTGTAGATGACCATGAGTC R: TCCTGCCAAACTTGCTCCAT	NM_031789.2	161
*HO-1*	F: ATGTCCCAGGATTTGTCCGA R: ATGGTACAAGGAGGCCATCA	NM_012580.2	144
*P62*	F: CTAGGCATCGAGGTTGACATT R: CTTGGCTGAGTACCACTCTTA	NM_175843.5	116
*LC3*	F: ACCAAGATCCCAGTGATTATAGAGC R: TTTTGCCTTGGTAGGGGCTT	NM_022867.2	340
*Beclin-1*	F: CAACCCCATGCTGTCCTTTC R: CTCTTGTCCCTTCCCCACAT	NM_053739.2	196
*β-Actin*	F: CAGCCTTCCTTCTTGGGTATG R: AGCTCAGTAACAGTCCGCCT	NM_031144.3	360

*Nrf2*: nuclear factor erythroid 2-related factor 2; *HO-1*: heme oxygenase-1; *P62* (SQSTM1): sequestosome 1, an autophagy adaptor protein involved in selective autophagy; *LC3*: microtubule-associated protein 1 light chain 3; *Beclin-1*: autophagy-related protein involved in autophagosome formation; *β-Actin*: housekeeping gene used as an internal control for normalization.

## Data Availability

The original contributions presented in this study are included in the article/Supplementary Materials. Further inquiries can be directed to the corresponding author.

## Supplementary Materials

The following supporting information can be downloaded at: https://www.mdpi.com/article/10.3390/ijms27135673/s1.

## Abbreviations

The following abbreviations are used in this manuscript:

ALT	Alanine aminotransferase
AFP	Alpha-fetoprotein
AST	Aspartate aminotransferase
Bax	Bcl-2-associated X protein
Bcl-2	B-cell lymphoma 2
CA19-9	Carbohydrate antigen 19-9
CA125	Cancer antigen 125
CA15-3	Cancer antigen 15-3
CAT	Catalase
CEA	Carcinoembryonic antigen
CD4	Cluster of differentiation 4
cDNA	Complementary deoxyribonucleic acid
DAB	3,3′-Diaminobenzidine
DCFH-DA	2′,7′-Dichlorodihydrofluorescein diacetate
DMH	1,2-Dimethylhydrazine
DNA	Deoxyribonucleic acid
EE%	Encapsulation efficiency
ELISA	Enzyme-linked immunosorbent assay
FTIR	Fourier transform infrared
GPx	Glutathione peroxidase
GSH	Reduced glutathione
H&E	Hematoxylin and eosin
HO-1	Heme oxygenase-1
HRP	Horseradish peroxidase
IHC	Immunohistochemistry
IL-1β	Interleukin-1 beta
IL-6	Interleukin-6
LC3	Microtubule-associated protein 1 light chain 3
MDA	Malondialdehyde
MIQE	Minimum information for publication of quantitative real-time PCR experiments
MPO	Myeloperoxidase
mRNA	Messenger ribonucleic acid
NADPH	Nicotinamide adenine dinucleotide phosphate (reduced form)
NF-κB	Nuclear factor kappa B
Nrf2/NRF2	Nuclear factor erythroid 2-related factor 2
OECD	Organization for Economic Co-operation and Development
PBS	Phosphate-buffered saline
PCR	Polymerase chain reaction
PDI	Polydispersity index
PLGA	Poly(lactic-co-glycolic acid)
PNL	Paeonol
PNL-PLGA	Paeonol-loaded poly(lactic-co-glycolic acid) nanoparticles
PVA	Polyvinyl alcohol
qPCR	Quantitative polymerase chain reaction
RFU	Relative fluorescence units
ROS	Reactive oxygen species
RT-PCR	Reverse transcription-polymerase chain reaction
SOD	Superoxide dismutase
TBARS	Thiobarbituric acid reactive substances
TEM	Transmission electron microscopy
TNF-α	Tumor necrosis factor-alpha
TP53	Tumor protein 53
8-OHdG	8-Hydroxy-2′-deoxyguanosine
