# Supplementary material for: Paeonol-Loaded PLGA Nanoparticles Attenuate DMH-Induced Colorectal Carcinogenesis-Associated Oxidative Stress, Inflammation, and Cellular Dysregulation via Modulation of NRF2/HO-1 Signaling in Rats
Source: Int J Mol Sci. 2026 Jun 23;27(13):5673. doi: 10.3390/ijms27135673 (PMC13361842; doi:10.3390/ijms27135673)
Supplement: Supplementary file 1 [file ijms-27-05673-s001.zip › ijms-4324906-supplementary.pdf]

Supplementary Materials

Table S1. Commercial kits and assay systems were used in the study.

Parameter/assay	Manufacturer	Country	Catalog number	Method/principle
Alanine aminotransferase (ALT)	Bio-Med Diagnostic	Egypt	MET-5123	Colorimetric / ELISA
Aspartate aminotransferase (AST)	Bio-Med Diagnostic	Egypt	MET-5127	Colorimetric / ELISA
Creatinine	Bio Diagnostic	Egypt	CR 12 51	Kinetic colorimetric
Urea	Bio Diagnostic	Egypt	UR 21 10	Urease–Berthelot colorimetric
Carcinoembryonic antigen (CEA)	MyBioSource	USA	MBS720630	Sandwich ELISA
Carbohydrate antigen 19-9 (CA19-9)	MyBioSource	USA	MBS729408	Sandwich ELISA
Cancer antigen 125 (CA125)	MyBioSource	USA	MBS732014	Sandwich ELISA
Cancer antigen 15-3 (CA15-3)	MyBioSource	USA	MBS2502096	Sandwich ELISA
Alpha-fetoprotein (AFP)	MyBioSource	USA	MBS054135	Sandwich ELISA
Reduced glutathione (GSH)	BioDiagnostic	Egypt	GR 25 11	Colorimetric
Glutathione peroxidase (GPx)	BioDiagnostic	Egypt	GP 25 24	Colorimetric
Catalase (CAT)	BioDiagnostic	Egypt	CA 25 17	Colorimetric
Superoxide dismutase (SOD)	BioDiagnostic	Egypt	SD 25 21	Colorimetric
Malondialdehyde (MDA)	BioDiagnostic	Egypt	MD 25 29	Thiobarbituric acid reactive substances (TBARS) colorimetric assay
Reactive oxygen species (ROS)	MyBioSource	USA	MBS2540517	Fluorometric assay
8-Hydroxy-2′-deoxyguanosine (8-OHdG)	MyBioSource	USA	MBS732375	ELISA

DNA fragmentation	MyBioSource	USA	MBS270898	ELISA
Tumor necrosis factor-alpha (TNF-α)	Assay Genie	Ireland	AEES00516	Sandwich ELISA
Interleukin-1 beta (IL-1β)	Assay Genie	Ireland	RTES00294	Sandwich ELISA
Interleukin-6 (IL-6)	Assay Genie	Ireland	RTFI00034	Sandwich ELISA
Nuclear factor kappa B (NF-κB)	MyBioSource	USA	MBS127898	ELISA
Cluster of differentiation 4 (CD4)	MyBioSource	USA	MBS70391	Sandwich ELISA
B-cell lymphoma 2 (Bcl-2)	MyBioSource	USA	MBS704498	ELISA
Bcl-2-associated X protein (Bax)	MyBioSource	USA	MBS2512405	ELISA
Caspase-3	MyBioSource	USA	MBS261814	ELISA
Tumor protein p53 (TP53)	MyBioSource	USA	MBS453009	ELISA

Abbreviations: ALT, alanine aminotransferase; AST, aspartate aminotransferase; CEA, carcinoembryonic antigen; CA19-9, carbohydrate antigen 19-9; CA125, cancer antigen 125; CA15-3, cancer antigen 15-3; AFP, alpha-fetoprotein; GSH, reduced glutathione; GPx, glutathione peroxidase; CAT, catalase; SOD, superoxide dismutase; MDA, malondialdehyde; TBARS, thiobarbituric acid reactive substances; ROS, reactive oxygen species; 8-OHdG, 8-hydroxy-2′-deoxyguanosine; TNF-α, tumor necrosis factor-alpha; IL-1β, interleukin-1 beta; IL-6, interleukin-6; NF-κB, nuclear factor kappa B; CD4, cluster of differentiation 4; TP53, tumor protein p53.
